# Supplementary material for: Towards precision psychiatry: Metabolomics identifies three biological subtypes of depression
Source: PLOS Digit Health. 2025 Dec 19;4(12):e0001125. doi: 10.1371/journal.pdig.0001125 (PMC12716697; doi:10.1371/journal.pdig.0001125)
Supplement: S1 File — Supplemental Method A. Genome-wide association study analysis. Supplemental Method B. Supplementary methods for sensitivity analysis. Fig A. QQ plots of GWAS analysis for the total depression sample and three depression subtypes compared to healthy controls. Fig B. Comparison of metabolic networks across depression subgroups. (DOCX) [file pdig.0001125.s001.docx]

Towards precision psychiatry: metabolomics identifies three biological subtypes of depression

*Supplemental Information S1 File*

# Supplemental Method A. Genome-wide association study analysis.

Approximately 488,000 participants in the UK Biobank cohort were genotyped using the Affymetrix UK BiLEVE Axiom or UK Biobank Axiom array. With a shared coverage of 95% between the two arrays, over 800,000 SNPs were genotyped. These genotype data were subsequently imputed and quality controlled against the Haplotype Reference Consortium (HRC) and UK10K haplotype resources, resulting in ~96 million imputed variants. Analysis in this study was conducted with version 3 of the UKBB imputed data, with 487,409 samples imputed and available for analysis following UKBB centrally performed QC filtering.

After excluding participants of non-White ancestry, high missingness, relatedness, discordant genetic and self-reported sex, and filtering SNPs based on minor allele frequency (MAF) ≥ 0.05 and imputation INFO score > 0.9, 105,044 samples were retained for subsequent analyses. All association tests were performed in PLINK v1.90b6.21 using logistic regression. The analysis was adjusted for age, sex, genotyping batch, genotyping array, and 5 population principal components.

To assess the impact of population stratification on the observed inflation of polygenic signals, linkage disequilibrium score regression (LDSC) was employed. Significant deviation of the intercept from 1 (±1.96 standard errors) was examined. The genomic inflation factor (λGC) was calculated to evaluate the deviation of the GWAS distribution from the null distribution.

Genetic correlations between depression subgroups and 34 diseases/traits were computed using linkage disequilibrium score regression (LDSR) analyses, implemented using the ‘ldsc’ package. GWAS summary statistics used in this study are available at https://zenodo.org/records/10515792 and GWAS Catalog. P-values were adjusted for multiple testing using Benjamini-Hochberg false discovery rate (FDR) correction.

# Supplemental Method B. Supplementary methods for sensitivity analysis.

Four additional sensitivity analyses were conducted in the UK Biobank dataset. These models sequentially excluded participants with immune-related diseases, metabolism-related diseases, those taking chronic medications, and those meeting all the above exclusion criteria.

(1) The definition of immune-related diseases was based on the study by Cai et al., as detailed in the table below [1].

| Immune-related diseases | ICD-10 codes |
| --- | --- |
| Asthma | J45, J46 |
| Rheumatoid arthritis Ulcerative colitis | M05, M06, M08 |
| Ulcerative colitis | K51 |
| Diabetes mellitus (Type I) | E10 |
| Rheumatic fever / rheumatic heart diseases | I00-I02, I05-I09 |
| Psoriasis | L40 |
| Celiac disease | K90.0 |
| Crohn's disease | K50 |
| Polymyalgia rheumatica | M35.3 |
| Multiple sclerosis | G35 |
| Allergic rhinitis | J301-304 |
| Rheumatism, unspecified | M79.0 |
| Psoriatic and enteropathic arthropathies | M07 |
| Graves' disease / Autoimmune thyroiditis | E05.0, E06.3 |
| Ankylosing spondylitis | M45 |
| Necrotizing vasculopathies | M31 |
| Sarcoidosis | D86 |
| Lichen planus | L43 |
| Sicca syndrome | M35.0 |
| Systemic Lupus erythematosus | L93, M32 |
| Idiopathic thrombocytopenic purpura | D69.3 |
| Primary biliary cholangitis | K74.3 |
| Myositis | M60 |
| Guillain-Barre syndrome | G61.0 |
| Myasthenia gravis | G70.0 |
| Bullous disorders | L10-L14 |
| Autoimmune hepatitis | K75.4 |

(2) The definition of metabolism-related diseases is provided in the table below.

| Metabolism-related diseases | ICD-10 (Field 41270) |
| --- | --- |
| Diabetes Mellitus | - E10 Insulin-dependent diabetes mellitus - E11 Non-insulin-dependent diabetes mellitus |
| Hypertensive diseases | - I10 Essential (primary) hypertension - I11 Hypertensive heart disease - I12 Hypertensive renal disease - I13 Hypertensive heart and renal disease - I15 Secondary hypertension |
| Heart diseases | - I20 Angina pectoris - I21 Acute myocardial infarction - I22 Subsequent myocardial infarction - I23 Certain current complications following acute myocardial infarction - I24 Other acute ischaemic heart diseases - I25 Chronic ischaemic heart disease |
| Renal failure | - N17 Acute renal failure - N18 Chronic renal failure - N19 Unspecified renal failure |
| Disorders of bone density and structure | - M80 Osteoporosis with pathological fracture - M81 Osteoporosis without pathological fracture - M82 Osteoporosis in diseases classified elsewhere - M83 Adult osteomalacia - M84 Disorders of continuity of bone - M85 Other disorders of bone density and structure |
| Endocrine, nutritional and metabolic diseases | - Disorders of thyroid gland - E10-E14 Diabetes mellitus - E15-E16 Other disorders of glucose regulation and pancreatic internal secretion - E20-E35 Disorders of other endocrine glands - E40-E46 Malnutrition - E50-E64 Other nutritional deficiencies - E65-E68 Obesity and other hyperalimentation - E70-E90 Metabolic disorders |
| Diseases of liver | - K70 Alcoholic liver disease - K71 Toxic liver disease - K72 Hepatic failure, not elsewhere classified - K73 Chronic hepatitis, not elsewhere classified - K74 Fibrosis and cirrhosis of liver - K75 Other inflammatory liver diseases - K76 Other diseases of liver - K77 Liver disorders in diseases classified elsewhere |
| Gout | - M10 Gout |
| Neoplasms | C00-C97  D00-D48 |

(3) Chronic medications included cholesterol-lowering agents, antihypertensive drugs, and insulin.

(4) The use of antidepressant and antipsychotic medications in the UK Biobank was defined based on the following fields:

| Treatment | Data Field | Data-Coding |
| --- | --- | --- |
| Antidepressant Usage | 20003 | 1140879616, 1140921600, 1140879540, 1140867878, 1140916282, 1140909806, 1140867888, 1141152732, 1141180212, 1140879634, 1140867876, 1140882236, 1141190158, 1141200564, 1140867726, 1140879620, 1140867818, 1140879630, 1140879628, 1141151946, 1140867948, 1140867624, 1140867756, 1140867884, 1141151978, 1141152736, 1141201834, 1140867690, 1140867640, 1140867920, 1140867850, 1140879544, 1141200570, 1140867934, 1140867758, 1140867914, 1140867820, 1141151982, 1140882244, 1140879556, 1140867852, 1140867860, 1140917460, 1140867938, 1140867856, 1140867922, 1140910820, 1140882312, 1140867944, 1140867784, 1140867812, 1140867668, 1140879624, 1140879632, 1140879552, 1140916288, 1140867916, 1140867806, 1140867824, 1140867712, 1140867720, 1140867722, 1140867734 |
| Antipsychotic Usage | 20003 | 1140868170, 1140928916, 1141152848, 1140867444, 1140879658, 1140868120, 1141153490, 1140867304, 1141152860, 1140867168, 1141195974, 1140867244, 1140867152, 1140909800, 1140867420, 1140879746, 1141177762, 1140867456, 1140867952, 1140867150, 1141167976, 1140882100, 1140867342, 1140863416, 1141202024, 1140882098, 1140867184, 1140867092, 1140882320, 1140910358, 1140867208, 1140909802, 1140867134, 1140867306, 1140867210, 1140867398, 1140867078, 1140867218, 1141201792, 1141200458, 1140867136, 1140879750, 1140867180, 1140867546, 1140928260, 1140927956 |

REFERENCES

1. Cai J, Rong H, Chen J, Deng Z, Chen S, Liao H, et al. Association of immune-mediated diseases with the risk of dementia and brain structure in UK Biobank participants. Age Ageing. 2024;53: afae274. doi:10.1093/ageing/afae274


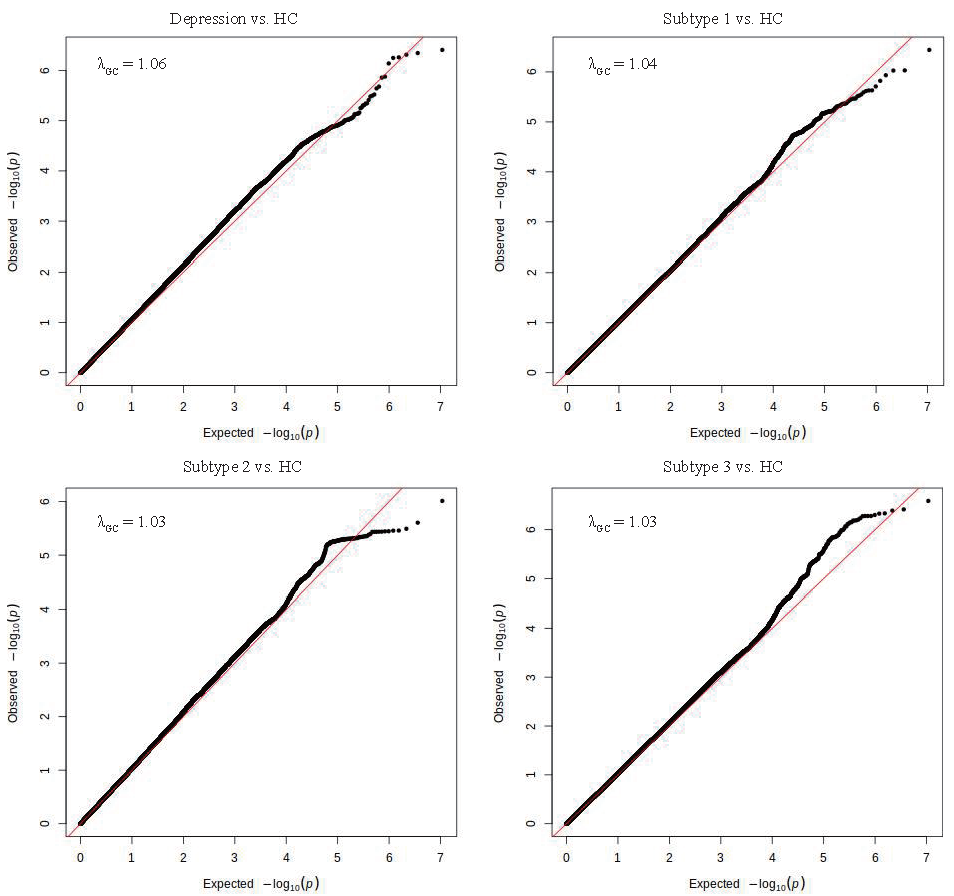


# Fig A. QQ plots of GWAS analysis for the total depression sample and three depression subtypes compared to healthy controls.


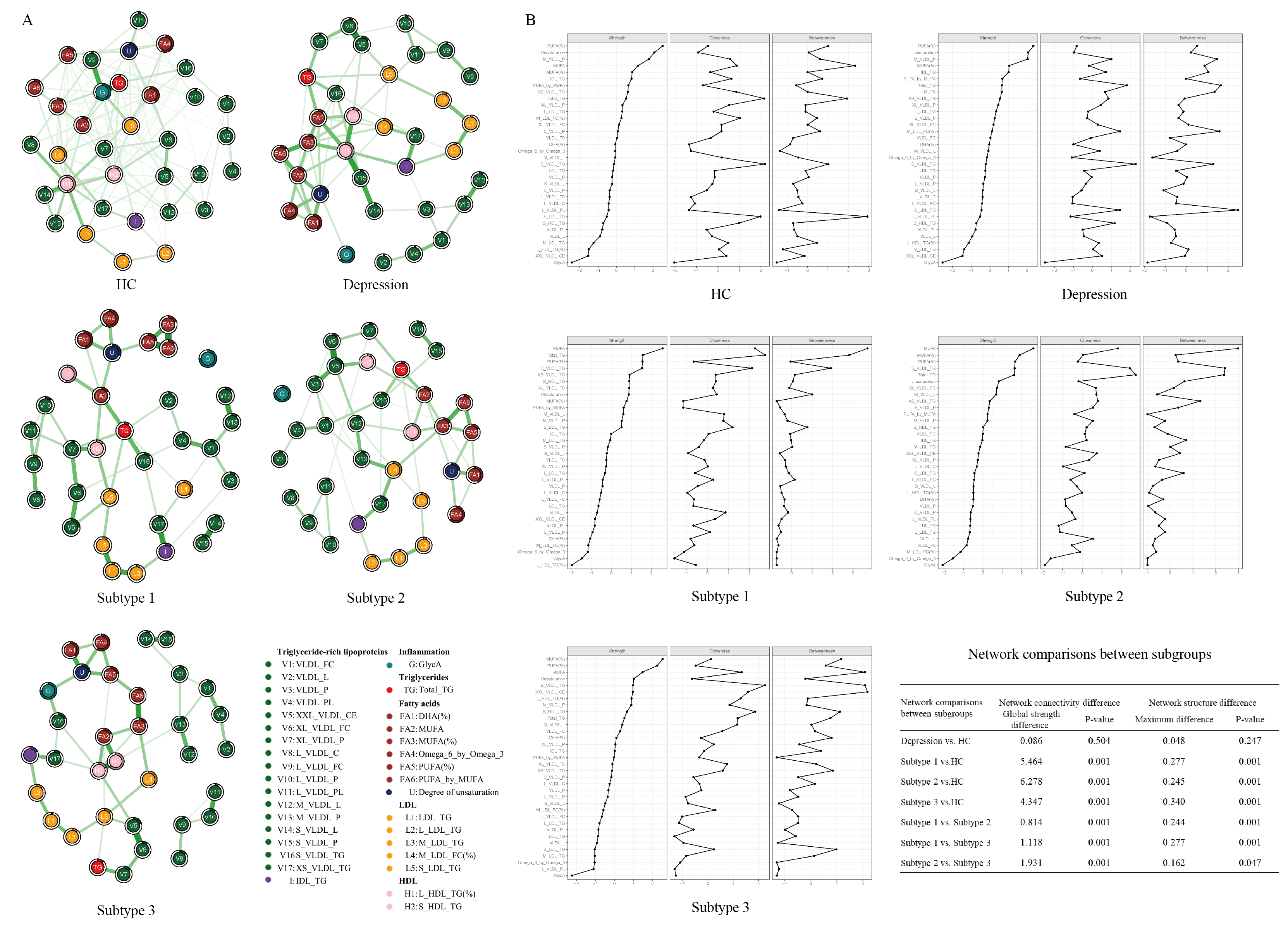


# Fig B. Comparison of metabolic networks across depression subgroups.

(1) Metabolic networks for each subgroup. (2) Comparison of centrality indices, global connectivity, and network structure among subgroups.
