# Supplementary material for: Towards precision psychiatry: Metabolomics identifies three biological subtypes of depression
Source: PLOS Digit Health. 2025 Dec 19;4(12):e0001125. doi: 10.1371/journal.pdig.0001125 (PMC12716697; doi:10.1371/journal.pdig.0001125)
Supplement: Attachment — Submitted filename: Response to reviewers.docx [file pdig.0001125.s004.docx]

Response to Reviewers：

We sincerely appreciate the time and effort you have dedicated to reviewing our manuscript. Your comments were immensely insightful and have greatly helped in improving the quality of our paper. In response to your suggestions, we have conducted extensive revisions to the previous draft. The key modifications are summarized below:

1. To enhance the robustness of our findings, we constructed four additional sensitivity analyses within the UK Biobank dataset. Specifically, we constructed four supplementary models that sequentially excluded the following groups of participants:

- those with immune-related diseases,
- those with metabolism-related diseases,
- those taking chronic medications, and
- those meeting all of the above criteria.

2. We have thoroughly reorganized the Discussion section, with particular emphasis on clarifying the clinical and biological implications of the identified subtypes, as well as elaborating on the limitations of our study.
3. In direct response to your comment, we have reanalyzed the medication usage data and included these updated results in the Supplementary Materials.

We believe that these revisions have substantially strengthened the manuscript and addressed the concerns you raised. Thank you once again for your constructive feedback.

**Reviewers' Comments:**

Reviewer #1: This is a well-designed and clinically relevant study that leverages metabolomic data from two large cohorts (UK Biobank and Whitehall II) to identify biologically distinct subtypes of depression. The identification of three metabolic subtypes—fatty acid dysregulated, hyperlipidemic, and intermediate—provides a valuable framework for advancing precision psychiatry. The study addresses a critical gap in depression research by moving beyond symptom-based classifications to biologically defined subtypes. This aligns with the NIH RDoC framework and could inform future targeted therapies. The study builds meaningfully on the authors’ prior work (Ma et al., 2024) by introducing clustering methods and improving diagnostic performance. While the work is strong, minor revisions would further strengthen its impact.
1. Please provide comparative analyses between depressed individuals and healthy controls (potentially as supplementary material) to assess whether these metabolic clusters are unique to depression or represent more general metabolic variations.
Response: We thank the reviewer for this suggestion. In response, we have included a comprehensive comparison of metabolic features between depression cases and healthy controls (HCs), as well as across different subtypes and HCs, in Supplementary Table 4.

The reason for not incorporating healthy controls in Figure 2E is primarily due to the large sample size of HCs, which would make the visualization visually overwhelming and difficult to interpret if displayed together with case subgroups. We aimed to maintain clarity in illustrating the metabolic differences among subtypes, and therefore focused on presenting the subtype-level patterns in the main figure.

2. A discussion of whether similar clustering patterns occur in other psychiatric disorders (e.g., bipolar disorder, anxiety disorders) or metabolic conditions (e.g., metabolic syndrome, diabetes) would help contextualize the findings. Are these patterns more strongly associated with depression-specific pathophysiological mechanisms (e.g., neuroinflammation, HPA axis dysregulation)?

Response: We thank the reviewer for this suggestion. Metabolic dysregulation is indeed a common feature across multiple psychiatric and metabolic disorders. However, the specific clustering pattern observed in our study—particularly the co-occurrence of fatty acid dysregulation, an intermediate phenotype, and hyperlipidemia—may reflect distinct pathophysiological processes specific to certain subgroups of depression. That said, these metabolic subtypes are unlikely to be exclusive to depression. They may instead represent perturbations of common biological pathways that manifest in different clinical contexts. For instance, the “hyperlipidemic” subtype may also be present in bipolar disorder with depressive episode, though their clinical implications and underlying mechanistic drivers may differ. Future studies directly comparing metabolic subtypes across psychiatric and metabolic disorders using high-throughput metabolomic profiling and consistent clustering methodologies are needed to disentangle disorder-specific biotypes from general transdiagnostic signatures. Such efforts could greatly advance the development of a biologically grounded nosology for mental disorders.

Based on this suggestion, we have revised the manuscript as follows:

The heterogeneity of depression has long impeded the discovery of biomarkers and the development of precise therapeutic strategies. This study demonstrates that stratifying patients into biologically homogeneous subtypes based on metabolomic profiles significantly enhances diagnostic precision. Unlike traditional binary classifications—which have shown limited utility in biomarker discovery—our approach identifies metabolically distinct subgroups, allowing for more accurate phenotypic differentiation. TRL and related lipoprotein components may help characterize specific metabolic subtypes of depression. As the terminal phase of systems biology research, metabolomics comprehensively reflects the dynamic changes of metabolites under pathophysiological conditions, thereby providing a critical biological basis for disease subtyping. Based on biologically defined subtypes derived from metabolomics, researchers can achieve finer classification of mental disorders and further integrate high-throughput multi-omics technologies—such as genomics, proteomics, and transcriptomics—to systematically elucidate underlying pathogenic mechanisms. This strategy is analogous to the clinical subcategorization of depression into subtypes such as “with anhedonia” and “without anhedonia,” aiming to uncover the molecular foundations of various subtypes and advance the implementation of individualized treatment and precision psychiatry. Future studies could explore whether these metabolic features contribute to subtype-specific diagnostic or intervention strategies, particularly when combined with other clinical and omics biomarkers. Indeed, metabolic disturbances are also commonly observed in other psychiatric disorders, such as schizophrenia, anxiety disorders, and bipolar disorder. Data-driven approaches could similarly be applied to identify biologically distinct subtypes within these conditions and promote transdiagnostic research in psychiatry. Most importantly, these metabolically defined subgroups open new avenues for targeted and subtype-specific interventions. By aligning treatment strategies with specific dysregulated metabolic pathways, we move closer to the vision of precision psychiatry—where therapy is tailored to an individual’s biological subtype. This represents a critical step forward from traditional one-size-fits-all diagnostic and therapeutic models.

While the stability of results after adjusting for sex and BMI is commendable, please discuss whether the observed metabolic changes might reflect secondary consequences of depression-related behaviors (e.g., altered appetite, physical activity) rather than primary disease mechanisms. For example, could the "hyperlipidemic" subtype represent metabolic changes associated with sedentary behavior rather than depression-specific biology?

Response: We have addressed this comment together with Question 5.
3. Please provide a more detailed characterization of the "intermediate" subtype. Is this a distinct biological group or more of a residual category? What are its defining metabolic or clinical features?
Response: The "intermediate" subtype identified in our analysis represents a metabolically transitional group, characterized by profiles that fall between the more extreme metabolic perturbations seen in the other two subtypes. Both metabolically and clinically, this subgroup exhibits features that are intermediate in severity and composition. We hypothesize that the intermediate subtype may reflect a heterogeneous mixture of individuals in transitional pathophysiological states. With larger sample sizes and additional high-dimensional biological data (e.g., proteomics or genomics), this group may be further resolved into two or more distinct subtypes. In the context of the current dataset, however, it most likely represents a clinically and biologically intermediate phenotype, rather than merely a residual category.
4. The manuscript should more explicitly highlight how the current clustering approach advances beyond the authors' previous machine learning analysis (Ma et al., 2024):
What specific improvements does the current methodology offer in terms of diagnostic accuracy, biological interpretability, or clinical utility?
Are there novel metabolic pathways identified in this study that were not apparent in the prior work?

Response: In our previous work, we adopted a supervised machine learning framework based on binary classification to identify depression-associated biomarkers, with a primary focus on diagnostic prediction. In contrast, the present study employs an unsupervised learning approach to uncover biologically distinct subtypes of depression. Unlike supervised learning, which relies on predefined labels for classification, unsupervised learning explores the inherent structure of the data, rendering it particularly suitable for identifying novel biological categorizations.

This shift in methodology resulted in a marked improvement in model performance. Specifically, subtype-based diagnostic models achieved a 10–40% increase in predictive accuracy compared to conventional binary classification. More importantly, we identified three metabolically heterogeneous subtypes: fatty acid dysregulation (Subtype 1), intermediate phenotype (Subtype 2), and hyperlipidemia (Subtype 3). Each subtype exhibits a unique pattern of metabolic disturbance associated with depression. These findings highlight the clinical and etiological significance of metabolic subtyping in depression and establish a foundation for future stratified research and personalized intervention strategies. That said, due to the relatively limited number of metabolomic features currently included, further work is needed to clarify the specific pathways characterizing each subtype. To systematically elucidate the underlying pathogenic mechanisms, subsequent studies should integrate high-throughput multi-omics technologies—such as genomics, proteomics, and transcriptomics—in a coordinated fashion.

5. The discussion should more carefully consider alternative interpretations of the metabolic findings: To what extent might the observed metabolic changes reflect lifestyle factors (e.g., diet, physical activity) rather than primary disease mechanisms?
While inflammatory markers (CRP, GlycA) and BMI differences are noted, please discuss whether these could be consequences rather than causes of the metabolic alterations.
These revisions would strengthen the manuscript's conclusions and provide a more nuanced interpretation of the important findings. The study represents a significant contribution to the field of precision psychiatry, and these suggested modifications would further enhance its impact.

Response: We thank the reviewer for this comment. In the revised manuscript, we have further addressed potential confounding influences on depression metabolism by conducting four additional sensitivity analyses. These analyses sequentially excluded individuals with: (1) immune-related diseases; (2) metabolism-related diseases; (3) those taking chronic medications; and (4) those meeting all of the above conditions. The results remained robust across all sensitivity models, supporting the stability of the metabolic subtypes identified.

We fully acknowledge that clarifying the causal relationship between the biological subtypes and lifestyle factors—such as diet, sedentary behavior, and physical activity—remains a limitation of this study. Regardless of whether metabolic alterations are a cause or consequence, subtyping provides a valuable framework for identifying high-risk individuals and guiding targeted interventions.

In response, we have added the following paragraph to the Discussion section to reflect these considerations:

Although the robustness of our model was maintained after adjusting for multiple potential confounders—including sex, BMI, chronic diseases, and medication use—it remains unclear whether the biological subtypes identified in this study, particularly the hyperlipidemic subtype, primarily reflect metabolic consequences of behavioral factors (such as sedentary behavior or poor dietary patterns) rather than depression-specific biological mechanisms. This important distinction warrants further investigation. Notably, the observed significant elevation of inflammatory markers within this subtype suggests that both biological and behavioral factors may interact in a bidirectional manner. Specifically, depressive symptoms may promote behavioral changes that exacerbate metabolic dysfunction, while metabolic alterations may in turn aggravate depressive symptoms through disruptions in energy homeostasis and neuroendocrine regulation. Given these complex interactions, future studies should explore whether tailored interventions—such as physical activity and dietary modifications—could serve as effective adjunctive therapies for specific depression subtypes, potentially mitigating both metabolic and mood-related symptoms.

Limitations: This study has several limitations that should be acknowledged. First, the cross-sectional design prevents us from establishing causal relationships between the identified metabolic subtypes and clinical outcomes. Although multiple sensitivity analyses were conducted, residual confounding from lifestyle factors—such as dietary habits and physical activity—cannot be fully ruled out. The observational nature of the data also limits our capacity to infer the direction of causality between metabolic dysregulation and depression. Future studies should adopt longitudinal designs and incorporate causal inference methods to verify the stability and biological foundations of these subtypes. Second, the scope of metabolomic coverage was limited by the number of metabolites available, which may affect the biological interpretability of the identified subtypes. We are currently addressing this issue in ongoing research through the use of high-throughput metabolomic platforms to uncover more precise and subtype-specific biomarkers. Third, although the UK Biobank currently offers the largest metabolomic dataset available, the dynamic characteristics of metabolic profiles necessitate careful consideration of disease state and sample collection timing. While the PHQ-2 was used for case identification, this instrument does not provide a comprehensive clinical assessment. Future work should employ more detailed clinical evaluations to better characterize subtype-specific symptomatology and its associations with metabolite profiles.

Reviewer #2: In this manuscript, the authors attempt to identify subtypes of depression using plasma metabolomics data from the UK Biobank and subsequently build machine learning-based diagnostic models. While the goal of advancing precision psychiatry is commendable, this study suffers from several critical and fundamental flaws in its design, subject selection, and data analysis. These issues are pervasive and significantly undermine the validity of the findings. In my assessment, these flaws are not correctable through revision, and therefore, I must recommend the rejection of this manuscript.
Below are my detailed comments outlining these major concerns.

Response: We thank the reviewer for their thorough and critical review of our manuscript. The points raised are indeed fundamental to the interpretation of our findings, and we have undertaken significant additional analyses to address them comprehensively.
Major Concerns:
1. Critical Bias in Patient and Control Selection: The methodology for cohort selection is fundamentally flawed.
1.1 Heterogeneous Definition of Depression: The authors define cases using UK Biobank Data-Field #130895, which includes both formal ICD-10 diagnoses and self-reported depression. Self-reports are not equivalent to a clinical diagnosis of Major Depressive Disorder (MDD) and may simply reflect depressive symptoms secondary to other conditions.

Response: We thank the reviewer for highlighting this important consideration, which we actively addressed in our initial study design. While the UK Biobank is not a depression-specific registry, it remains the largest source of publicly accessible metabolomic data. In this exploratory study of depression subtypes, we therefore relied on the composite definition of depression provided in UK Biobank Data-Field 130895. We fully acknowledge that this approach does not equate to a clinical diagnosis of Major Depressive Disorder (MDD), and have consistently used the broader term “depression” rather than “MDD” throughout the article for this reason. To increase diagnostic confidence, we also imposed an additional inclusion criterion of PHQ-2 > 2.

A Venn diagram was created to visualize the overlap between depression cases identified through ICD codes and those based on self-report (Figure X). We recognize that gold-standard MDD diagnosis requires clinical validation, typically available only in dedicated prospective studies; however, no large-scale metabolomic database currently exists with exclusively clinically verified MDD cases. Given this limitation, we used the best available population-level data, following methodologies well-established in prior literature including Fleetwood et al. (PLOS Medicine, 2025) and Chen et al. (BMC Medicine, 2024).

Figure 1. Sources of Depression Diagnosis.
1.2 Insufficient Exclusion Criteria: The manuscript states that the exclusion criteria include a history of schizophrenia or bipolar disorder. However, it fails to exclude patients with other major medical or psychiatric conditions that are known to profoundly alter metabolic profiles. For example, patients with a primary diagnosis of cancer, autoimmune disorders, or other chronic inflammatory diseases may be included in the depression cohort, making it impossible to disentangle the metabolic signature of depression from that of the comorbidity. The authors also did not exclude patients with anxiety disorders, a common comorbidity that shares metabolic and inflammatory pathways with depression.

Response: We thank the reviewer for this comment. We would like to clarify that participants with cancer were in fact excluded based on our initial inclusion criteria. Furthermore, in direct response to the reviewer’s concern regarding the potential confounding effect of comorbid immune diseases, we have now incorporated an additional sensitivity analysis in the revised manuscript, in which we excluded all individuals with immune-related diseases. The specific criteria used to define immune-related diseases are provided below. The definition of immune-related diseases was based on the classification established by Cai et al. in *Age and Ageing* (2024), as detailed in the table below.

Table 1. The definition of immune-related diseases.

| **Immune-related diseases** | **ICD-10 codes** |
| --- | --- |
| Asthma | J45, J46 |
| Rheumatoid arthritis Ulcerative colitis | M05, M06, M08 |
| Ulcerative colitis | K51 |
| Diabetes mellitus (Type I) | E10 |
| Rheumatic fever / rheumatic heart diseases | I00-I02, I05-I09 |
| Psoriasis | L40 |
| Celiac disease | K90.0 |
| Crohn's disease | K50 |
| Polymyalgia rheumatica | M35.3 |
| Multiple sclerosis | G35 |
| Allergic rhinitis | J301-304 |
| Rheumatism, unspecified | M79.0 |
| Psoriatic and enteropathic arthropathies | M07 |
| Graves' disease / Autoimmune thyroiditis | E05.0, E06.3 |
| Ankylosing spondylitis | M45 |
| Necrotizing vasculopathies | M31 |
| Sarcoidosis | D86 |
| Lichen planus | L43 |
| Sicca syndrome | M35.0 |
| Systemic Lupus erythematosus | L93, M32 |
| Idiopathic thrombocytopenic purpura | D69.3 |
| Primary biliary cholangitis | K74.3 |
| Myositis | M60 |
| Guillain-Barre syndrome | G61.0 |
| Myasthenia gravis | G70.0 |
| Bullous disorders | L10-L14 |
| Autoimmune hepatitis | K75.4 |

After excluding all participants with immune-related diseases, the performance of our diagnostic model remained robust. Compared to the traditional binary classification model, the AUC increased by 10.1% to 39.6% (Figure 2).

Figure 2. Diagnostic performance of the model after excluding participants with immune-related diseases.

We have consulted with other psychiatrists and concluded that it would not be appropriate to completely exclude individuals with anxiety disorders. This is primarily because depression and anxiety disorders are highly comorbid in clinical practice, exhibit substantial symptomatic overlap, and share common neurobiological mechanisms—such as HPA-axis dysregulation and inflammatory activation—making it difficult to separate the two conditions in real-world populations. Excluding individuals with anxiety would result in an artificially “pure” cohort that fails to reflect clinical reality, where mixed anxiety-depression presentations are common. Retaining these patients enhances the generalizability and clinical relevance of our findings.

1.3 Lack of Comorbidity Analysis: The study fails to stratify or at least compare the distribution of major comorbidities (e.g., cancer, cardiovascular disease) across the identified subtypes. This is a significant omission, as the "hyperlipidemia" subtype, for instance, could simply be a cluster of patients with metabolic syndrome rather than a true biological subtype of depression.
Response: Thank you for your suggestion. Similar to the previous point, we would like to clarify that participants with cancer had already been excluded based on our initial inclusion criteria. In direct response to your comment, we have now conducted an additional sensitivity analysis by further excluding individuals with common metabolism-related diseases, as detailed in the table below. Following this exclusion, the model's performance improved notably, with the AUC increasing by 11.5% to 40.4% (Figure 3). The diagnostic performance remained robust, indicating that the biological subtypes we identified are highly stable and reliable.

Table 2. The definition of metabolism-related diseases.

| **Metabolism-related diseases** | **ICD-10 (Field 41270)** |
| --- | --- |
| Diabetes Mellitus | - E10 Insulin-dependent diabetes mellitus - E11 Non-insulin-dependent diabetes mellitus |
| Hypertensive diseases | - I10 Essential (primary) hypertension - I11 Hypertensive heart disease - I12 Hypertensive renal disease - I13 Hypertensive heart and renal disease - I15 Secondary hypertension |
| Heart diseases | - I20 Angina pectoris - I21 Acute myocardial infarction - I22 Subsequent myocardial infarction - I23 Certain current complications following acute myocardial infarction - I24 Other acute ischaemic heart diseases - I25 Chronic ischaemic heart disease |
| Renal failure | - N17 Acute renal failure - N18 Chronic renal failure - N19 Unspecified renal failure |
| Disorders of bone density and structure | - M80 Osteoporosis with pathological fracture - M81 Osteoporosis without pathological fracture - M82 Osteoporosis in diseases classified elsewhere - M83 Adult osteomalacia - M84 Disorders of continuity of bone - M85 Other disorders of bone density and structure |
| Endocrine, nutritional and metabolic diseases | - Disorders of thyroid gland - E10-E14 Diabetes mellitus - E15-E16 Other disorders of glucose regulation and pancreatic internal secretion - E20-E35 Disorders of other endocrine glands - E40-E46 Malnutrition - E50-E64 Other nutritional deficiencies - E65-E68 Obesity and other hyperalimentation - E70-E90 Metabolic disorders |
| Diseases of liver | - K70 Alcoholic liver disease - K71 Toxic liver disease - K72 Hepatic failure, not elsewhere classified - K73 Chronic hepatitis, not elsewhere classified - K74 Fibrosis and cirrhosis of liver - K75 Other inflammatory liver diseases - K76 Other diseases of liver - K77 Liver disorders in diseases classified elsewhere |
| Gout | - M10 Gout |
| Neoplasms | C00-C97  D00-D48 |

Figure 3. Diagnostic performance of the model after excluding participants with metabolism-related diseases.
2. Lack of Temporal Alignment Between Diagnosis and Blood draw for Metabolomics: A crucial flaw is the failure to ensure that the metabolomics data were collected during a current depressive episode that was diagnosed prior to the blood draw. The Methods section notes an exclusion for those "diagnosed with depression after the baseline assessment," but the blood samples for metabolomics were collected over a wide window (2019-2022). The manuscript does not confirm that for each patient, the blood draw date was cross-referenced with the diagnosis date (Field #130896) to establish a clear temporal link. Without this confirmation, the metabolic state measured may precede the onset of depression or occur long after remission, rendering the entire analysis invalid for identifying state-dependent biomarkers.
Response: Thank you for raising this important point, which we may not have articulated clearly enough in the main text. We sincerely appreciate the opportunity to clarify.

The blood samples used for metabolomic profiling in UK Biobank were collected at the baseline assessment visit for all participants. Our case definition required that a diagnosis of depression be recorded prior to this baseline visit, and that the participant also had a PHQ-2 score > 2 at baseline. This approach ensures a temporal sequence in which the diagnosis precedes the blood draw, directly addressing the reviewer’s concern regarding chronology.

The metabolomics measurements were done later (in 2019–2022), but they were done on the blood that was taken at the beginning. This means the metabolic results still reflect people’s health at the time they were diagnosed.

We have updated the Methods section to explain this more clearly.
3. Inherent Bias in Metabolomics Data: The study's conclusions are constrained by the severe limitations of the UK Biobank's metabolomics panel.
3.1 Limited and Biased Coverage: The panel measures only 249 metabolic indicators, which represents a tiny fraction (< 0.2%) of the known human metabolome (over 20,000).
3.2 Overrepresentation of Lipids: Critically, the panel is heavily biased towards lipid metabolism. As the authors note, 210 of the 249 markers are related to lipoprotein lipids and another 18 are fatty acids. With ~92% of the input data related to lipids, it is statistically inevitable that any clustering algorithm would identify subtypes based on lipid dysregulation. The study, therefore, does not discover novel biological subtypes so much as it confirms the input bias of its dataset. The "fatty acid dysregulation" and "hyperlipidemia" subtypes are likely artifacts of the measurement panel rather than distinct depression biotypes.

Response: Thank you for raising this point, which we fully acknowledge as a limitation of our study and have accordingly addressed in the Limitations section of our manuscript.

While high-throughput metabolomics is a powerful tool for biomarker discovery in depression, the exploration of biologically distinct subtypes requires particularly large and rich datasets to ensure that the identified subgroups are both statistically robust and clinically meaningful. We recognize that the UK Biobank metabolomics data, despite its biases, currently represents the most comprehensive resource available for this purpose, especially when compared to smaller-scale studies.

In this work, by identifying preliminary metabolic subtypes of depression, we aim to provide a foundation for more targeted subsequent research. For example, we are already conducting follow-up studies in our lab focusing on targeted fatty acid and cholesterol-related metabolites. We strongly believe that further validation and refinement of these subtypes will depend on collaborative efforts to build large, multi-omics datasets specifically designed for psychiatric research moving forward.

Moreover, we agree that the biological subtypes identified here may not be unique to depression and could potentially extend to other psychiatric disorders. Nevertheless, the underlying pathogenic mechanisms are likely to differ across diagnostic boundaries. Building on the subtypes delineated in this work, we encourage future studies to integrate additional high-throughput technologies—such as genomics, proteomics, and transcriptomics—to systematically unravel the distinct molecular mechanisms driving each subtype and ultimately advance the field toward precision psychiatry.
4. Uncontrolled Confounding Variables (For example the use of antidepressants and statins): The analysis fails to account for key confounders that directly influence metabolic profiles. Most notably, the use of antidepressant medications, statins, or other psychotropic and metabolic drugs was not compared between subtypes or controlled for in the analysis. These medications have well-documented effects on lipid, fatty acid, and inflammatory markers, and their differential use among patients could easily explain the metabolic differences observed between the proposed subtypes.

Response: Thank you very much for this suggestion. We have addressed it in the revised manuscript.

To evaluate the potential impact of commonly used chronic medications—including cholesterol-lowering agents, antihypertensive drugs, and insulin—on our results, we performed a sensitivity analysis that excluded individuals using these medications. The results showed that, even after this exclusion, the AUC increased by 12.6% to 40.3% compared to the binary classification model, underscoring the robustness of our findings (Figure 4).

Additionally, to account for the collective influence of immune-related diseases, metabolism-related diseases, and chronic medications, we conducted a fourth sensitivity analysis that simultaneously excluded all individuals with any of these conditions. Reassuringly, the model continued to demonstrate improved performance, with the AUC increasing by 13.6% to 41.9%, further supporting the stability and reliability of the identified metabolic subtypes (Figure 5).

We also provided a statistical summary of the use of antidepressant and antipsychotic medications across the subtypes in the Supplementary Materials. The proportions of antidepressant use in Subtypes 1, 2, and 3 were 59.85%, 64.37%, and 69.32%, respectively, while the corresponding proportions of antipsychotic use were 1.81%, 2.57%, and 3.72%. Significant differences were observed among the three subgroups. These findings are consistent with the PHQ-4 and PHQ-2 scores, which also showed a graded increase in depressive symptom severity across the subtypes, with Subtype 3 > Subtype 2 > Subtype 1.

Figure 4. Diagnostic performance of the model after excluding participants taking chronic medications.

Figure 5. Diagnostic performance of the model after excluding participants with immune-related diseases, metabolism-related diseases, and chronic medications.
5. Flawed Machine Learning Methodology: The approach to building and evaluating the diagnostic models is unsatisfactory and lacks rigor.
5.1 Handling of Imbalanced Data: The dataset is extremely imbalanced, with 7,945 depression cases compared to over 175,000 healthy controls. The Methods section makes no mention of how this severe class imbalance was handled. Standard machine learning practice requires techniques like oversampling (e.g., SMOTE), undersampling (e.g., ENN), or a hybrid approach (e.g., SMOTE-ENN) to prevent the model from simply learning to predict the majority class (healthy controls). This omission is a critical methodological flaw that invalidates the reported performance metrics (AUCs).

5.2 Unsatisfactory Model Performance and Selection: While the subtype models show higher AUCs than the binary model, the performance for Subtype 1 (AUC 0.785) and Subtype 2 (AUC 0.817) is modest and may be inflated due to the data imbalance issue. Furthermore, the reliance on a single algorithm (CatBoost) is not well-justified. A robust study would involve testing and optimizing a suite of models, including potentially more powerful deep learning architectures designed for tabular data (e.g., FT-Transformer, GANDALF), to ensure the best possible model is selected.

Response: Response: Thank you for raising this important methodological point. We appreciate the opportunity to clarify our approach to handling class imbalance.

In our analysis, we directly addressed the class imbalance issue within the CatBoost algorithm by setting the class_weights parameter to a vector inversely proportional to class frequencies. This method effectively increases the weight of the minority class (depression cases) within the loss function. As a result, during training, the model assigns a higher penalty for misclassifying minority-class samples, which shifts the decision boundary to improve recognition of the underrepresented class. This approach allows the model to achieve more balanced recall without altering the original data distribution through oversampling or undersampling techniques. We have updated the Methods section to include a clearer description of this weighting strategy.

CatBoost was selected for building the diagnostic model in this study based on the following considerations: First, as a tree-based ensemble method, CatBoost demonstrates excellent performance in handling structured biomedical data, particularly in scenarios involving class imbalance and a large number of categorical features—such as those commonly encountered in UK Biobank data. Our prior research also indicated that, among multiple machine learning models, CatBoost achieved optimal performance for this type of task. More importantly, the primary objective of this study was not to maximize diagnostic AUC performance, but rather to identify and validate potential metabolic subtypes within depressive disorders. Here, the machine learning model serves an exploratory and auxiliary role, aimed at evaluating the discriminative feasibility of these subtypes rather than conducting systematic algorithmic comparisons. Therefore, multiple models were not introduced for benchmarking. We acknowledge that although the AUC values for the identified metabolic subtypes (e.g., Subtype 1 and Subtype 2) did not exceed 0.90, they still achieved approximately 10%–40% improvement compared to traditional binary classification models. This enhancement carries potential clinical significance, suggesting that metabolic subtypes can provide more biologically informative distinctions than a unified disease label.

Future work may incorporate a wider range of machine learning or deep learning models to further optimize classification performance. However, the current study focuses on establishing a novel metabolic subtyping framework to elucidate heterogeneity in depression.
Minor Concerns:
1. Missing Methodological Details: Figure 2 presents visualizations using UMAP and t-SNE. However, these dimensionality reduction techniques are not described anywhere in the Methods section.

Response: We thank the reviewer for this comment. In response, we have now supplemented the Methods section with a detailed description of our dimensionality reduction and visualization approach. Specifically, we applied Non-negative Matrix Factorization (NMF) for dimensionality reduction, followed by visualization of the resulting low-dimensional representations using both Uniform Manifold Approximation and Projection (UMAP) and t-Distributed Stochastic Neighbor Embedding (t-SNE) to illustrate potential cluster structures in a low-dimensional space.
2. Ambiguity of Control Group: It is not explicitly clear whether the same healthy control (HC) group was used for all comparisons against the different depression subtypes in the various analyses (GWAS, machine learning, etc.). This should be clarified.

Response: Thank you for your comment. Regarding the GWAS analysis, the same set of healthy controls (HCs) was used consistently across all comparative analyses. However, for the machine learning component, the control group for each subtype-specific diagnostic model was constructed by including all participants not belonging to that particular subtype. For example, the model for Subtype 1 was trained using Subtype 1 cases versus controls consisting of HCs, Subtype 2, and Subtype 3 participants. This approach has been clarified in the revised manuscript.

Reviewer #3: The study employed various methods, including biological assessment, cohort study, and machine learning analysis, to provide valuable insights into specific biomarkers of depression. Providing data on depression subtype and its implications for intervention is valuable and practical. the study used large sample of UK biobank data for analysis which made it more rigorous study.
Some points should be considered for further clarification as follows:
1. In the introduction section, the field of metabolomics study has been compared with binary, categorical, and diagnostic. It would be more appropriate to discuss a dimensional approach to diagnosis and treatment, comparing it with the main approach of the current study.

Response: We thank the reviewer for this constructive suggestion. We have revised the manuscript accordingly to address this point. The modified content now reads as follows:

The heterogeneity of depression has long impeded the discovery of biomarkers and the development of precise therapeutic strategies. This study demonstrates that stratifying patients into biologically homogeneous subtypes based on metabolomic profiles significantly enhances diagnostic precision. Unlike traditional binary classifications—which have shown limited utility in biomarker discovery—our approach identifies metabolically distinct subgroups, allowing for more accurate phenotypic differentiation. TRL and related lipoprotein components may help characterize specific metabolic subtypes of depression. As the terminal phase of systems biology research, metabolomics comprehensively reflects the dynamic changes of metabolites under pathophysiological conditions, thereby providing a critical biological basis for disease subtyping. Based on biologically defined subtypes derived from metabolomics, researchers can achieve finer classification of mental disorders and further integrate high-throughput multi-omics technologies—such as genomics, proteomics, and transcriptomics—to systematically elucidate underlying pathogenic mechanisms. This strategy is analogous to the clinical subcategorization of depression into subtypes such as “with anhedonia” and “without anhedonia,” aiming to uncover the molecular foundations of various subtypes and advance the implementation of individualized treatment and precision psychiatry. Future studies could explore whether these metabolic features contribute to subtype-specific diagnostic or intervention strategies, particularly when combined with other clinical and omics biomarkers. Indeed, metabolic disturbances are also commonly observed in other psychiatric disorders, such as schizophrenia, anxiety disorders, and bipolar disorder [1–3]. Data-driven approaches could similarly be applied to identify biologically distinct subtypes within these conditions and promote transdiagnostic research in psychiatry. Most importantly, these metabolically defined subgroups open new avenues for targeted and subtype-specific interventions. By aligning treatment strategies with specific dysregulated metabolic pathways, we move closer to the vision of precision psychiatry—where therapy is tailored to an individual’s biological subtype. This represents a critical step forward from traditional one-size-fits-all diagnostic and therapeutic models.
2. In the introduction ref #12, a study on the UK Biobank sample is mentioned. The biomarkers achieved an AUC diagnostic score of 0.6-0.7, which is insufficient for accurate diagnosis. How it could be justified and explained

Response: Thank you for raising this point. The study referenced as #12, published by our team in 2024, indeed demonstrated that conventional binary classification models for depression—using metabolomic data alongside demographic variables—achieved a maximum AUC of 71.6%. Models based solely on traditional risk factors reached an AUC of 71.1%, while those using metabolomic features alone attained an AUC of 62.4%. These results confirm that relying exclusively on metabolomic markers provides limited diagnostic utility in clinical settings.

It was precisely these limitations that motivated the current study. Rather than continuing with conventional binary classification, we adopted a data-driven approach to identify biologically distinct subtypes of depression within the metabolomic data. This strategy allowed us to account for the inherent heterogeneity of depression and ultimately significantly improve diagnostic performance in a subtype-specific manner, as reflected in the elevated AUC values presented in this work.
3. Why did the "methodology" section come after the discussion section?

Response: We appreciate the reviewer’s attention to manuscript structure. The placement of the Methods section after the Discussion section aligns with the specific formatting guidelines of this journal, which encourages a focus on key findings and narrative continuity in certain types of articles.
4. What exactly is the meaning of "depression": minor, major, dysthymia …. Type of depression? Why has PHQ-2 instead of PHQ-9 been used for case finding? The enrolled cases may not be considered as "major depressive disorder". Major depressive disorder, as mentioned by the article, is a heterogeneous disorder, which should not be considered an exact binary diagnosis by using precise diagnostic criteria.

Response: In this study, cases of depression were identified based on the UK Biobank’s composite definition, which incorporates diagnostic information from four sources: death registers, primary care records, hospital admissions data, and self-reported diagnoses, mapped to corresponding ICD-10 codes. Specifically, we included cases coded under ICD-10 F32 (depressive episode). As this broad definition does not align strictly with the clinical concept of Major Depressive Disorder (MDD), we consistently use the term “depression” throughout the manuscript.

To ensure a meaningful temporal association between depression status and metabolomic markers, we required that all cases must have received a diagnosis prior to the baseline assessment and exhibited a PHQ-2 score > 2 at baseline. Blood samples used for metabolomic profiling were also collected during the baseline visit.

Although rigorously phenotyped cohorts often rely on verified clinical diagnoses, the scale and design of the UK Biobank necessitate a more inclusive case definition.

Moreover, since the full PHQ-9 was not administered at UK Biobank baseline—only the first four items were available—we relied on the well-validated abbreviated measure PHQ-2, with a threshold of > 2, as a proxy for depressive symptoms. This cutoff is supported by previous literature as an effective screening tool for depression in large-scale epidemiological contexts, justifying its use in our study.

5. This study helps to make a subtype of depressive disorder and has clinical implications; however, it does not add value to accurate diagnosis, as written in lines 273-274. How could it be justified?

Response: We thank the reviewer for their comment. We believe there may be a misunderstanding regarding the diagnostic improvement achieved in our study.

All primary and sensitivity analyses demonstrated that the subtype-based diagnostic models achieved a 10–40% increase in AUC compared to the conventional binary classification model. Certain features, such as triglyceride-rich lipoproteins (TRL), contributed to even greater improvements in specific models.

Furthermore, to evaluate the potential confounding effects of immune-related diseases, metabolism-related disorders, and commonly used medications for chronic conditions, we conducted additional sensitivity analyses. The results remained robust, consistently showing significantly enhanced performance relative to the binary baseline. Thus, our findings support the conclusion that metabolic subtyping improves the discriminative capacity of diagnostic models for depression beyond what is achievable with a unitary case-control framework.
6. "...This suggests that future research could develop streamlined diagnostic models for depression utilizing TRL and different lipoprotein components…" 281-282 LINES, again, do not explain the biomarker for diagnosis of depression. TRL may be involved in many normal and abnormal biological processes, it may just provide a feature of MDD. As mentioned in ref 33, the metabolic network associated with specific depressive symptoms may have intervention implications.

Response: We thank the reviewer for this insightful comment. We agree that TRL and other lipoproteins are involved in numerous biological processes and are not specific to depression. Our intention was not to propose them as standalone diagnostic biomarkers, but rather to suggest that they may serve as key discriminative features within a larger metabolomic signature associated with specific depressive subtypes.

We have revised the manuscript as follows:

TRL and related lipoprotein components may help characterize specific metabolic subtypes of depression. As the terminal phase of systems biology research, metabolomics comprehensively reflects the dynamic changes of metabolites under pathophysiological conditions, thereby providing a critical biological basis for disease subtyping. Based on biologically defined subtypes derived from metabolomics, researchers can achieve finer classification of mental disorders and further integrate high-throughput multi-omics technologies—such as genomics, proteomics, and transcriptomics—to systematically elucidate underlying pathogenic mechanisms. This strategy is analogous to the clinical subcategorization of depression into subtypes such as “with anhedonia” and “without anhedonia,” aiming to uncover the molecular foundations of various subtypes and advance the implementation of individualized treatment and precision psychiatry. Future studies could explore whether these metabolic features contribute to subtype-specific diagnostic or intervention strategies, particularly when combined with other clinical and omics biomarkers.
